# Recovery process of vertical perception and activities of daily living in stroke patients: A retrospective cohort study

**DOI:** 10.1002/brb3.3001

**Published:** 2023-04-11

**Authors:** Kota Sawa, Kazu Amimoto, Keisuke Ishigami, Takuya Miyamoto, Chika Ishii, Rikuya Suzuki, Miko Tamura, Akira Morizane, Chikashi Komatsu, Mitsusuke Miyagami

**Affiliations:** ^1^ Department of Physical Therapy, Faculty of Health Sciences Ryotokuji University Urayasu Chiba Japan; ^2^ Department of Physiotherapy, Graduate School of Human Health Sciences Tokyo Metropolitan University Arakawa‐ku Tokyo Japan; ^3^ Department of Rehabilitation Takenotsuka Noshinkei Rehabilitation Hospital Adachi‐ku Tokyo Japan; ^4^ Department of Rehabilitation Tokyo Sakura Hospital Edogawa‐ku Tokyo Japan

**Keywords:** activities of daily living, stroke, subjective postural vertical

## Abstract

**Introduction:**

Clarifications regarding the recovery process of the subjective postural vertical (SPV) and activities of daily living in stroke patients are required to help clinicians determine treatment plans. Therefore, we aimed to investigate the characteristics of the longitudinal recovery process of SPV and activities of daily living after stroke.

**Methods:**

Overall, 109 patients with stroke were enrolled. Clinical assessments included the SPV and total functional independence measure (FIM), initially and after 1 month. The mean and standard deviation of SPV indicated the directional and variability errors, respectively. Participants were categorized as follows: nondeviation group comprised directional and variability errors within the standard values, deviation of variability errors group comprised directional errors within the standard value and variability errors greater than the standard value, and deviation of both directional and variability errors group comprised directional and variability errors greater than the standard values. In addition, a two‐way analysis of variance was performed for initial pre‐ and post‐SPV, and pre‐ and posttotal FIM scores (*p *< .05).

**Results:**

The deviation of variability errors group, and deviation of both directional and variability errors group, had larger SPV variability errors than did the nondeviation group. Furthermore, the deviation of variability errors group showed a significant improvement in variability errors after 1 month. There was a correlation between the initial SPV with eyes opened variability error and total FIM after 1 month in Pusher patients with unilateral spatial neglect in the deviation of both directional and variability errors group.

**Conclusions:**

SPV with eyes opened variability errors and initial FIM score may influence the independence of activities of daily living after 1 month in the recovery of patients with stroke with Pusher and unilateral spatial neglect.

## Graphical abstract

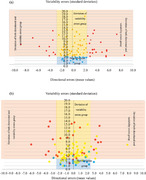

## 1 INTRODUCTION

The number of strokes recorded in 2017 reached 1.12 million in Japan, in which is the highest rates (422/100,000 person‐years among men and 212/100,000 person‐years among women) (Venketasubramanian et al., 2017). According to the 2021 population census, the leading causes of death in Japan are malignant neoplasms, cardiac disease, age‐associated cognitive decline, and stroke. However, dementia ranks first among people certified as needing long‐term care, followed by stroke, cognitive impairment associated with aging, and fractures and falls. Stroke rehabilitation plays an important role in the Japan's aging population and it is associated with a high social burden.

Postural control is affected by visual, vestibular, and somatosensory perceptions and influences balance (Horak, 2006). The capability to maintain equilibrium usually refers to the functioning of the trigeminal nerve via a pathway that involves the cochlea and otoliths, among others; meanwhile, postural control refers to the body's capability to maintain stability via somatosensory and neural networks (Horak, 2006). Balance disorders in stroke patients are associated with perceptual dysfunction. Cognitive impairment inhibits walking independence (Karatas et al., 2004), which is required to maintain activities of daily living (ADLs) (Barra et al., 2007; Verheyden et al., 2006). Many factors affect balance, including somatic and higher cognitive functions, which are disrupted in sensory impairment and subjective postural vertical (SPV) disorders (Karnath et al., 2000). Age, motor function, and other present and past diseases have also been implicated in balance dysfunction. This study aimed to evaluate the impact of SPV on ADLs, whereby SPV was conceptualized as a higher brain function and was disrupted in stroke patients with balance impairment.

Vertical perception (VP), which is involved in postural control (Bisdorff et al., 1996), affects the body's ability to sit and stand (Pérennou et al., 2000). Impaired VP results in delayed poststroke recovery of function and ADL (Bonan et al., 2006), and it can be used as a proxy for measuring balance (Dai et al., 2021; Yelnik et al., 2002). VP includes the experiences described as subjective visual vertical (SVV), SPV, and subjective postural vertical with eyes opened (SPV‐EO). Pérennou et al. (2008) reported that the normal range of directional errors between SVV and SPV was ±2.5°. In addition, Karatas et al. (2004) reported that the normal range of SPV variability errors was within 3.3°. Pusher behavior (PB) and unilateral spatial neglect (USN) are among the poor prognostic factors for ADLs; they are associated with delayed recovery. Meanwhile, SPV is involved in gravity perception and should be evaluated as prerequisite for balance and a factor affecting PB. We have previously shown that SPV may influence ADLs (Sawa et al., 2022). PB with USN after stroke show deviations in the mean (directional errors) and standard deviation (variability errors) of SPV values (Barra et al., 2010, 2009; Davies, 1985; Karnath et al., 2000; Pérennou et al., 2008; Saj et al., 2005). Karnath et al. (2000) reported that the SPV of patients with Pusher syndrome was deflected by 17.9° to the nonparetic side. The main mechanism of PB was the deflection of VP owing to the impairment of gravity perception, resulting in pushing or resisting postural correction (Davies, 1985; Karnath et al., 2000). SPV deviations occurred in PB‐only cases, and SPV‐EO variability errors increased and delayed recovery in the process for ADLs in cases with USN (Sawa et al., 2022). Karnath et al. (2000) reported that the SPV deviated to the nonparetic side in PB cases, and the variability errors of SPV‐EO, rather than the SPV deviation, increased in USN cases. People affected by PB and USN experience delayed recovery in a manner similar to that observed in hemiplegic cases. However, factors that affect recovery in PB and USN remain unclear. Sue et al. (2022) investigated the differences in the clinical evaluation of 19 patients with Pusher syndrome after stroke by categorizing them into two groups according to the degree of brain damage and studying their progress over 1‐year in a retrospective cohort study. The group with a lesion score of ≥2 had a significantly delayed recovery from PB, and the hazard ratio of the preexisting brain lesion score was 0.458 (95% confidence interval: 0.221, 0.949), while the side of hemiparesis was not a significant covariate. In addition, old age and severe impairments were associated with delayed recovery from lateropulsion (LP) in a manner specific to the side of the lesion, contraversive pushing in patients with motor and functional deficits (Dai et al., 2021; Pérennou et al., 2008).

Barra et al. (2007) conducted a longitudinal study on LP to examine the course of standing posture control, balance, and gait. Recently, LP with contra‐lesion is the primary factor altering poststroke balance and gait in the subacute stage; therefore, it should be systematically assessed (Dai et al., 2021). Poststroke balance and gait rehabilitation should incorporate techniques devoted to misorientation with respect to gravity. The scale for contraversive pushing (SCP) cutoff values calculated from the Youden Index were 3.5 for balance and 1 for gait.

It was reported that patients with Pusher syndrome had increased variability when the mean value of SPV measurement was used as the directional errors and the standard deviation as the variability errors. Recent systematic reviews suggested that longitudinal analysis and characterization of pathophysiology in verticality such as SPV was important for prognostication (Cian, 2017; Riberio et al., 2021). The prognosis of ADLs recovery may differ between patients with increased directional and variability errors; however, the factors that contribute to this difference are unclear. Furthermore, the recovery pathways of patients with PB and USN with directional or fluctuating errors are unclear. Therefore, by examining how SPV or SPV‐EO affects ADLs, and identifying factors that contribute to this outcome, we can improve our understanding of the recovery pathway of patients with Pusher syndrome. Moreover, the Stroke Recovery and Rehabilitation Roundtable guideline recommend that prognostication be performed early in the diagnosis and treatment process, so consensus can be reached on rehabilitation. Herein, we collected data at one at diagnosis and 1 month thereafter, as these are critical moments in the patient journey.

This study used multiple regression analysis to determine factors that affect SPV and FIM after 1 month. The factors that affect ADLs recovery in Pusher syndrome, and their relationship to SPV, remain unclear. This study aimed to identify factors relevant in the prognostication of ADLs recovery by dividing patients into subgroups that were followed up longitudinally.

This is the first longitudinal study on the relationship between SPV and ADLs recovery in stroke and Pusher syndrome patients. This study examined longitudinal changes in the relationships among the key variables.

## 2 METHODS

### 2.1 Participants

**FIGURE 1:**
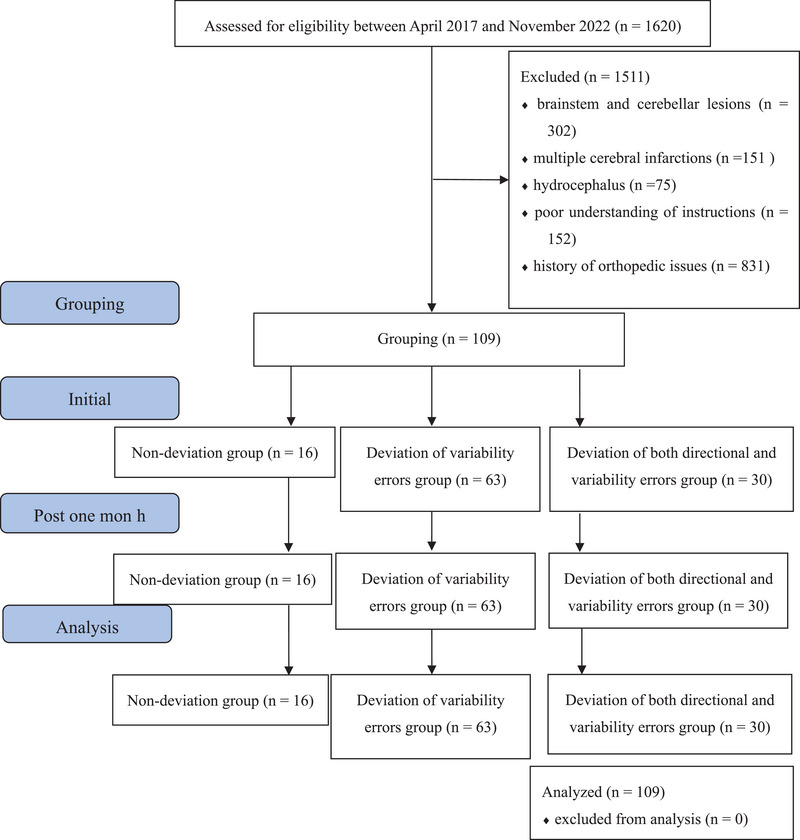
Strengthening the reporting of observational studies in epidemiology flow diagram of the study. Nondeviation group: patients whose mean initial SPV measurements (directional errors) were within −2.5° and +2.5° and the standard deviations (variability errors) did not deviate from ≤3.3°. Deviation of variability errors group: patients whose mean initial SPV measurements were within −2.5° and +2.5° and the variability errors were > 3.3°. Deviation of both directional and variability errors group: patients whose initial SPV measurements deviated from the range of −2.5° to +2.5° and the variability errors were > 3.3°. SPV, subjective postural vertical.

Among the 1620 patients with stroke admitted to the Takenotsuka Noshinkei Rehabilitation Hospital between April 2017 and November 2022, 109 patients with VP measurements were enrolled in this retrospective cohort study after applying the inclusion and exclusion criteria (Figure 1).

The inclusion criteria were first‐ever stroke with a unilateral lesion and being right‐handed. The exclusion criteria were brainstem and cerebellar lesions, multiple cerebral infarctions, hydrocephalus, poor understanding of instructions, and orthopedic musculoskeletal findings, such as fractures, rheumatism, and cases in which pain was clinically judged by a physician to interfere with ADLs. This study adhered to the principles of the Declaration of Helsinki and Strengthening the Reporting of Observational Studies in Epidemiology Statement guidelines. It was approved by the Sonodakai Ethics Committee (No. 141) and University Hospital Medical Information Network Center (No 000049806) and all participants provided written informed consent.

### 2.2 Grouping

Patients were grouped as follows: the nondeviation group consisted of patients whose mean initial SPV measurements (directional errors) were within −2.5° and +2.5° (Pérennou et al., 2000), and the standard deviations (variability errors) did not deviate from ≤3.3° (Fukata et al., 2020); the deviation of variability errors group consisted of patients whose mean initial SPV measurements were within −2.5° and +2.5° and whose variability errors were > 3.3°; the deviation of both directional and variability errors group consisted of patients whose initial SPV measurements deviated from the range of −2.5° to +2.5° and whose variability errors were >3.3° (Figure 1).

### 2.3 Demographics and clinical assessments

Demographic and clinical data were obtained from patients’ medical records (Table 1). Demographic data included age, sex, number of days between onset and measurement, diagnosis, lesion, lesion side, Brunnstrom recovery stage (BRS), and stroke impairment assessment set (SIAS) score at admission. Regarding the clinical assessments, the initial pre‐SPV, post‐SPV (1 month after pre‐SPV assessment), initial pretotal functional independence measure (FIM) score, and posttotal FIM score (1 month after pre‐FIM assessment) were retrieved from the medical records.

**TABLE 1:** Demographic characteristics

Group (ANOVA)	Age^a^	From onset^a^	Days on SPV from onset^a^	Damage side^a^	Type^a^	Region^b^	Sex^a^	Pusher (SCP)^c^ USN (BIT)^c^	Frequency of occurrence of pusher behavior^c^	Aphasia^b^	BRS (lower)^c^
*p* Value	.801	.412	.705	.175	.811	Group A: .094 Group B: .000 Group C: .539	.805	Pusher (SCP): .883 USN (BIT): .735	.883	.30	.721
*F* value	0.222	0.893	0.351	1.775	0.210	–	0.217	Pusher (SCP): 0.914 USN (BIT): 0.313	0.914	–	1.122
Group A (*n* = 16)	68.3 ± 10.9 years	21.5 ± 11.9 days	43.4 ± 20.3 days	Right: 8 Left: 8	CI: 8 CH: 8	Ic1, C1, Ins1, O1, TPJ1, Par1, PF2, Th2, Pu6	Men: 11 Women: 5	Pusher: 3 (3.8) USN: 2 (98.5)	18.8%	3	I: 2 II: 6 III: 2 IV: 3 V: 1 VI: 2
Group B (*n* = 63)	70.6 ± 12.1 years	21.8 ± 12.4 days	43.6 ± 35.0 days	Right: 44 Left: 19	CI: 27 CH: 36	Ic4, C1, Ins2, M15, S15, O3, TPJ3, PF9, Th19*, Pu12*	Men: 40 Women: 23	Pusher: 17 (4.7) USN: 19 (76.1)	27.0%	8	I: 5 II: 18 III: 13 IV: 14 V: 10 VI: 3
Group C (*n* = 30)	70.2 ± 14.1 years	24.8 ± 11.2 days	49.2 ± 26.9 days	Right: 23 Left: 7	CI: 12 CH: 18	Ic3, C3, Ins2, M11, S12, O3, TPJ1, Par4, PF1, Th4, Pu6	Men: 21 Women: 9	Pusher: 14 (5.1) USN: 14 (60.8)	46.7%*	5	I: 3 II: 16 III: 3 IV: 3 V: 3 VI: 2

Group A, nondeviation group; Group B, deviation of variability errors group; Group C, deviation of both directional and variability errors group; SPV, subjective postural vertical; SCP, scale for contraversive pushing; USN, unilateral spatial neglect; BIT, Behavioral Inattention Test; BRS, Brunnstrom recovery stage; SLF, superior longitudinal fasciculus; Ic, internal capsule; S, striatum; C, corona radiata; Ins, insula; Par, parietal cortex; T, temporal cortex; TPJ, temporoparietal junction; O, occipital cortex; PF, prefrontal cortex; M1, primary motor cortex; S1, primary sensory cortex; Th, thalamus; Pu, Putamen.

^a^ One‐way analysis of variance (ANOVA).

^b^ Chi‐square test.

^c^ Mann‒Whitney *U* test, *p* < .05.

* Significant difference, *p* < .05.

### 2.4 Measurement of subjective postural vertical values

**FIGURE 2:**
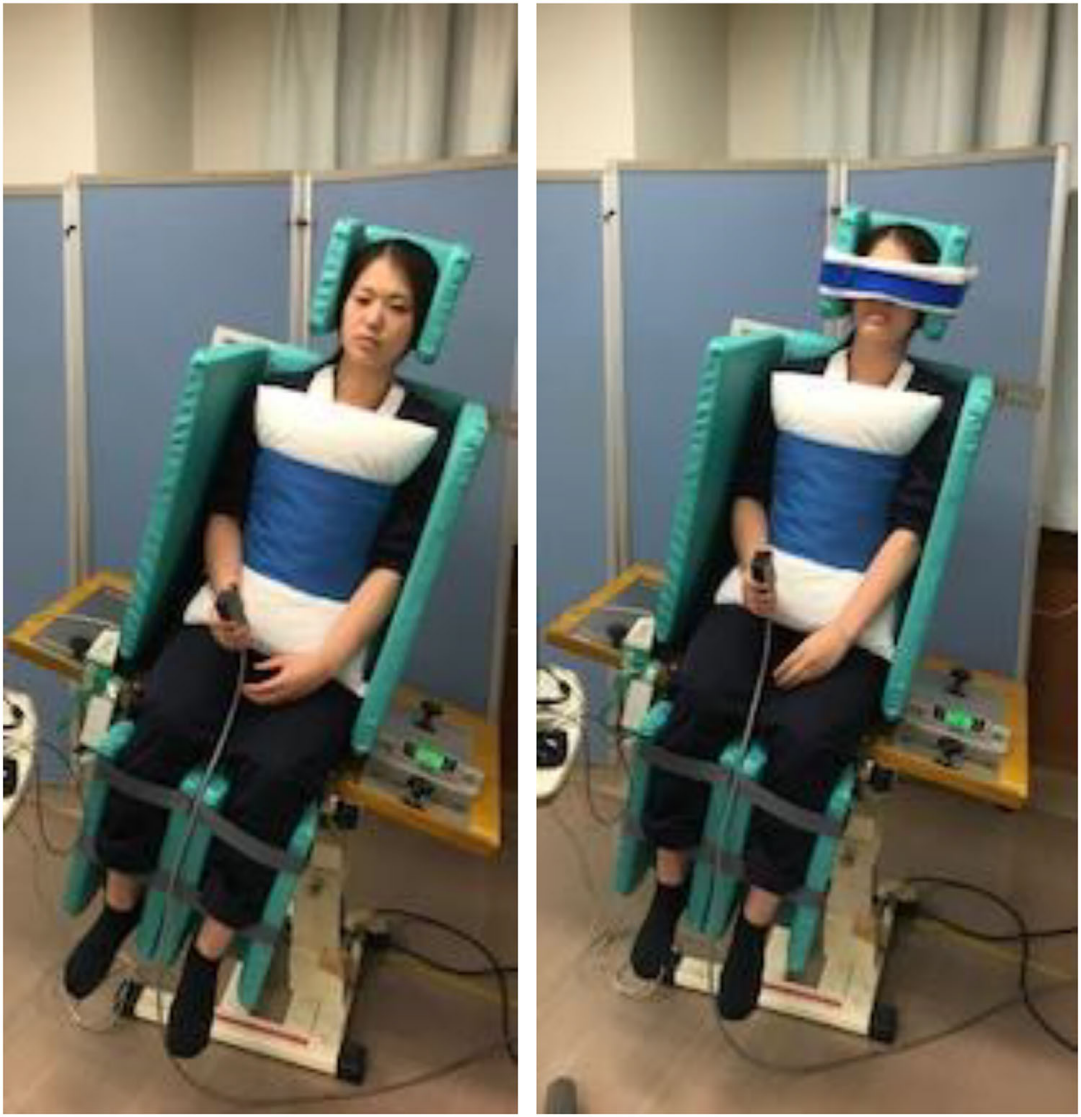
Subjective postural vertical and subjective postural vertical with eyes opened measurement on the automatic vertical board.

SPV values were measured using a motorized vertical cognitive tilt device (Pair Support, Inc., Saitama, Japan) (Figure 2). Regarding the practice test, we evaluated the patient's arousal, so that it would not affect the measurement, and we conducted the assessment only when the patient could understand verbal instructions. The test was conducted in a quiet environment in a rehabilitation room, and an eye mask was used when the SPV eyes were closed. The room was separated so that the surroundings were not crowded, and the conditions in the room were kept constant. SPV was measured at the point where each participant considered themselves sitting vertically relative to their trunk line. The participants sat on the device and were inclined at 15‒20° to the left or right at 1.5° per second. The SPV measurements were performed in a randomized order. The starting direction of the tilt measurements was left or right, based on the ABBABAAB or BAABABBA sequences. The standard deviation of eight measurements was used as the PV tilt variability (Fukata et al., 2020; Sawa et al., 2022).

The total FIM is an ADLs assessment battery consisting of 18 items (13 motor and five cognitive items), with 18 points being the lowest score (severe) and 126 points being the highest score (good) (Granger et al., 1986).

### 2.5 Assessments of pusher behavior and unilateral spatial neglect

PB was assessed using the SCP (Karnath et al., 2000), which includes the following variables: postural asymmetry, abduction, or extension of the nonparetic extremities, and resistance to passive postural correction. Each item was scored from 0 to 2, which were summed to yield a total score ranging from 0 to 6; a score of six indicated severe PB, which was further specified by a score >0 for each item and a total SCP score of 1.75 points according to the cutoff established by Baccini et al. (2008). The SCP was assessed by a physical therapist. We did not perform this test as an ocular or spatial coordinate evaluation; we performed the USN evaluation as a pathological examination. USN was assessed using the Behavioral Inattention Test conventional subtest (BITC) (Ishiai, 1999), which included the following six items: line crossing, letter cancellation, star cancellation, figure and shape copying, line bisection, and representational drawing. The BITC score ranges from 0 to 146 points, with a score of ≤131 points indicating USN. BITC was conducted in a quiet room with an area of 7.81 m^2^ by an occupational or speech therapist. During the test, participants sat on chairs with backrests, with their feet on the ground.

### 2.6 Statistical analyses

Demographic data were compared between the two groups using the one‐way analysis of variance (ANOVA) with the Bonferroni post hoc test, chi‐square test, and Mann–Whitney *U* test. The mean and standard deviation of the SPV measurements were used as the directional and variability errors, respectively. The SPV directional and variability errors for initial pre‐ and postdata, and the initial pre‐ and posttotal FIM scores were compared using ANOVA with the Bonferroni post hoc test, respectively. In addition, the relationship between the change in VP and total FIM in the deviation of variability errors group and in the deviation of both directional and variability errors group were assessed using Pearson's correlation coefficients. For all tests, *p *< .05 was considered statistically significant.

Statistical analysis was performed using multiple regression analysis with total FIM at 1 month as the dependent variable. Independent variables were as follows: age, sex, Mini‐Mental State Examination test findings, number of days from onset to SPV measurement, injured hemisphere side, BRS of the lower limb, SIAS, Behavioral Inattention Test (BIT), SCP, initial SPV, SPV‐EO directional errors, initial SPV, and SPV‐EO variability errors (*p <* .05).

A sample size of 106 cases was determined using a two‐way ANOVA and the Bonferroni post hoc test, with an *α* of 0.05, *p *< .05, power of 0.8, and effect size (*f*) of 0.35 (G*power version 3.1.1, Tokyo, Japan).

## 3 RESULTS

### 3.1 Demographic data

Sixteen, 63, and 30 patients were classified into all groups, respectively. No significant differences in age, sex, days from the onset of SPV measurement, BRS, and the Pusher and USN severity were observed in each group (Table 1). In this study, no participant died or dropped out during the follow‐up period from post‐1 month.

### 3.2 Changes in subjective postural vertical directional errors

**FIGURE 3:**
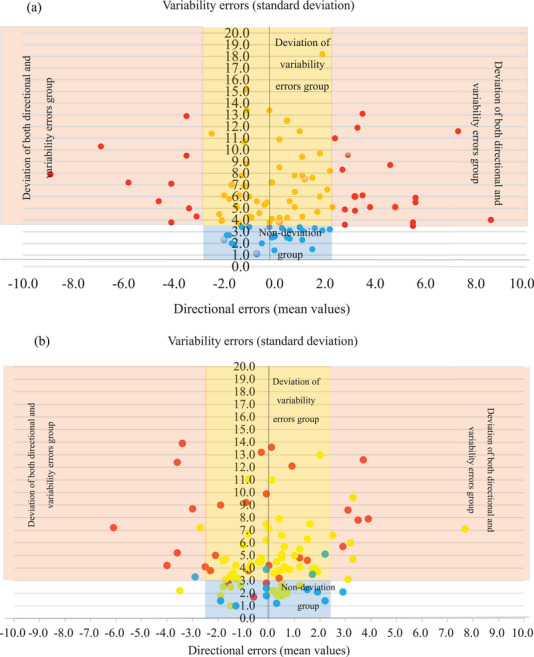
Subjective postural vertical directional and variability errors. (a) Initial subjective postural vertical directional and variability errors. (b) Postsubjective postural vertical directional and variability errors. Nondeviation group: Subjective postural vertical directional errors within −2.5° to 2.5° and subjective postural vertical variability errors within 3.3° (*n* = 16). Deviation of variability errors group: Subjective postural vertical directional errors within −2.5° to 2.5° and subjective postural vertical variability errors over 3.3° (*n* = 63). Deviation of both directional and variability errors group: Subjective postural vertical directional errors outside the range −2.5° to 2.5° and subjective postural vertical variability errors without 3.3° (*n* = 29).

No significant differences in the group mean of SPV directional errors were observed between the groups (Table 2, Figure 3a and b).

**TABLE 2:** Subjective postural vertical directional and SPV variability errors

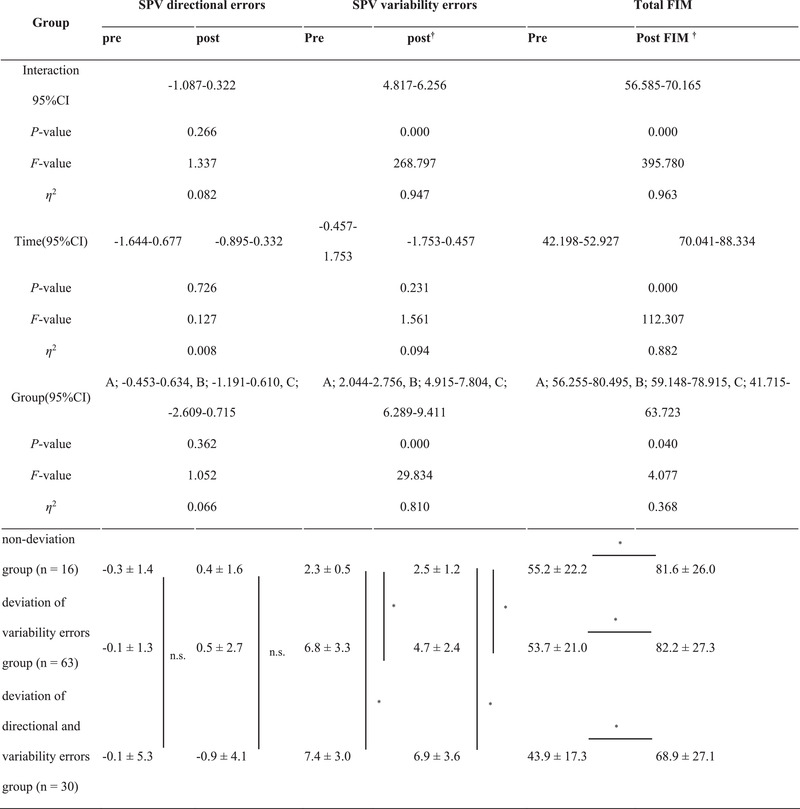

Group A, nondeviation group; Group B, deviation of variability errors group; Group C, deviation of both directional and variability errors group; SPV, subjective postural vertical; FIM, functional independence measure; n.s., no significant difference.

^†^ Interactions (group, time), *p *< .05.

* Simple main effects (group), *p *< .05.

### 3.3 Changes in subjective postural vertical variability errors

Regarding SPV variability errors, a significant difference in time was observed, with interaction and simple main effects by the groups; the deviation of variability errors group, and deviation of both directional and variability errors group, scored higher than did the nondeviation group. In addition, after 1 month, the values were higher in the deviation of variability errors group than in the nondeviation group and higher in the deviation of both directional and variability errors group than in the deviation of variability errors group (Table 2, Figure 3a and b).

### 3.4 Changes in the total functional independence measure score

A simple main effect of timing and interaction between timing and group in all groups were found. Additionally, an improvement in the FIM score at 1 month was also observed (Table 2).

### 3.5 Pusher characteristics in nondeviation group, deviation of variability errors group, and deviation of both directional and variability errors

Three, 17, and 14 Pusher cases were classified into all groups, respectively. The results of the intergroup comparison showed no significant differences in the severity of Pusher cases, USN severity, initial SPV directional errors, initial SPV variability errors, and initial FIM score. However, no significant differences in the SPV directional errors and FIM score were observed at 1 month (Table 3).

**TABLE 3:** Pusher behavior and unilateral spatial neglect characteristics in all groups

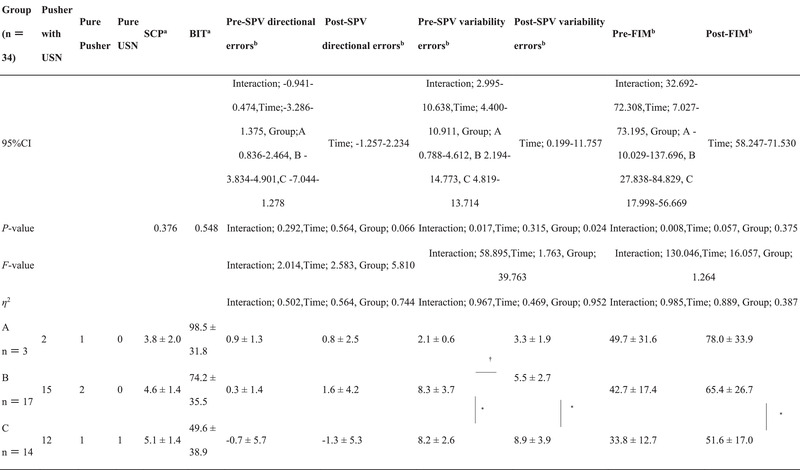

Group A, nondeviation group; Group B, deviation of variability errors group; Group C, deviation of both directional and variability errors group; SCP, scale for contraversive pushing; BIT, Behavioral Inattention Test; SPV, subjective postural vertical; FIM, functional independence measure.

^a^ Mann–Whitney *U* test.

^b^ Two‐way ANOVA.

^†^ Interactions (group, time), *p *< .05.

* Simple main effects (group), *p *< .05.

### 3.6 Factors associated with postfunctional independence measure in deviation of variability errors group and deviation of directional and variability errors group

The correlation between post‐FIM and initial SPV variability errors (*r *= −0.57, *p *< .05) and initial FIM were moderately positive (*r *= 0.87, *p *< .01) in the deviation of both directional and variability errors group, but not in the deviation of variability errors group (Table 4).

**TABLE 4:** Relationships between postfunctional independence measure and subjective postural vertical values with eyes opened and prefunctional independence measure after stroke with Pusher behavior and unilateral spatial neglect in deviation of variability errors group and deviation of both directional and variability errors group

Post‐FIM	Group B (*n* = 14) C (*n* = 17)	Age	Sex	Days from onset	Damage side	BRS	MMSE	SCP	BIT	SIAS	Pre‐SPV‐EO directional errors	Pre‐SPV directional errors	Pre‐SPV‐EO variability errors	Pre‐SPV variability errors	Pre‐FIM
Spearman correlation coefficients	B C	0.15 0.31	–0.27 −0.09	–0.23 −0.28	0.27 −0.01	0.15 −0.04	–0.42 −0.04	0.25 −0.45	0.19 0.64	–0.17 −0.18	0.04 −0.31	0.27 0.04	–0.41 −0.57*	0.15 −0.12	0.62** 0.87**
*p* Value	B C	.58 .29	.30 .76	.37 .33	.29 .98	.58 .90	.12 .91	.34 .11	.56 .09	.52 .55	.89 .28	.29 .89	.10 .04	.58 .68	.01 .00

Group B, deviation of variability errors group; Group C, deviation of directional and variability errors group; FIM, functional independence measure; BRS, Brunnstrom recovery stage; MMSE, Mini‐Mental State Examination; SCP, scale for contraversive pushing; BIT, Behavioral Inattention Test; SIAS, stroke impairment assessment set; SPV, subjective postural vertical; SPV‐EO, subjective postural vertical with eyes opened.

* Significant difference, *p <* .05.

** Significant difference, *p <* .01.

### 3.7 Predicted variables of the recovery process in patients with Pusher syndrome

In the multiple regression analysis, total initial FIM and initial SPV variability were errors related to patients with Pusher syndrome in all groups (Table 5).

**TABLE 5:** Prognosis factors in postfunctional independence measure of pusher behavior and unilateral spatial neglect in groups nondeviation group, deviation of variability errors group and deviation of both directional and variability errors group (*n* = 34)

	B	Standard error	β	*F* value	*t*‐value	*p* Value	*R*	*R* ^2^	95%CI
Post‐FIM = initial FIM + initial‐SPV‐EO variability errors	45.492	10.229	–	22.838	4.447	.00	0.72	0.60	24.631 to 66.354
SPV‐EO variability errors	–2.412	0.867	–0.338	–	–2.782	.009			–4.181to –0.644
Pre‐FIM	0.769	0.159	0.588	–	4.838	.000			0.445 to 1.093

FIM, functional independence measure; SPV‐EO, subjective postural vertical with eyes opened.

The predicted equation was as follows: post‐FIM = 0.769 initial FIM + (−2.412) initial SPV‐EO variability errors + 45.492(*R = *0.72, *R* ^2^ *= *0.60, *F* value* = *22.838, *p = *.00).

## 4 DISCUSSION

This study investigated the recovery of SPV and total FIM after 1 month, based on the SPV classification in 109 patients. Specifically, we first used a two‐way analysis of variance to determine whether the subgroups were making good progress after 1 month. Next, correlation analysis was performed to examine the impact of SPV on FIM. Finally, the impact of subgroup C on the FIM of Pusher syndrome patients was investigated.

Longitudinal evidence on SPV is lacking, including the associated parameters relevant to prognostication (Cian, 2017; Riberio et al., 2021), and this study aimed to address this gap in knowledge. Herein, patients in the deviation of variability errors group demonstrated the most significant improvements in SPV variability errors and total FIM after 1 month. In the deviation of variability errors group, patients with Pusher syndrome with SPV directional errors within the normal range showed improved variability errors and FIM after 1 month. Moreover, neither interactions nor main effects were observed in any group. This finding may be due to the fact that the SPV directional errors of the nondeviation group and the deviation of variability errors group were within the normal range. Regarding the SPV variability errors, simple main effects were detected. In the recovery of SPV variability errors, the initial SPV and the SPV after 1 month were within the normal range in the nondeviation group. In contrast, the deviation of variability errors group showed the greatest improvement in SPV variability errors. This is because the initial SPV in the deviation of variability errors group was significantly affected by the starting point of the measurement procedure (Fukata et al., 2017). However, in the deviation of both directional and variability errors group, the SPV directional errors were biased from the beginning, and the standard deviation around the mean value was also significant. The measurements were affected by the starting position and basic axis of the deviated VP.

Previous studies (Sawa et al., 2022; Fukata et al., 2020; Karnath et al., 2000) have reported that the VP of patients with Pusher syndrome was biased toward the nonparetic or paretic side. Moreover, bilateral bias existed even in healthy participants (Baggio et al., 2016; Fujino et al., 2017). In this study, the VP of participants was biased toward the nonparetic or paretic side, which may have prevented the deviation of the total mean value.

Fujino et al. (2017) reported that the minimal detectable change with 95% confidence (MDC_95_) in the SPVs of healthy participants was 1.2°. Similarly, in this study, the improvement in SPV variability errors was 2.1° after 1 month in the deviation of variability errors group, which is above the MDC_95_, indicating that true improvement in SPV variability errors occurred only in the deviation of variability errors group after 1 month.

Regarding the clinical assessment, no differences in PB and USN severity were observed between the deviation of variability errors group and deviation of both directional and variability errors group in the initial evaluation; however, SPV variability errors and total FIM improved after 1 month in the deviation of variability errors group. These results suggest that the prognosis of patients with unbiased directional errors in the deviation of variability errors group was better than that of patients in the deviation of both directional and variability errors group during the recovery process after stroke.

An interaction effect between time and group and a simple main effect of time were observed in all groups. In particular, the interaction between time and SPV classification contributed to the improvement of the total FIM in the deviation of variability errors group. Beninato et al. (2006) found that the cutoff value of the minimal clinically important difference (MCID) for improving total FIM after stroke was 3.0. With a positive ratio of 3.3, in a patient with total FIM changes by 22 points, the probability of achieving the MCID is 96% (Beninato et al., 2006), suggesting that the total FIM change in group B exceeded the MCID. Therefore, it may be possible to predict the prognosis based on the characteristics of the change in total FIM after 1 month by examining whether the first or second SPV is within the normal range. Moreover, SPV‐EO to improve stroke prognosis, and furthermore, to influence the course of FIM. This suggested that there was an effect of improvement in ADLs.

No significant differences between the deviation of variability errors group and deviation of both directional and variability errors group regarding the basic characteristics and PB severity were observed; however, the SPV variability errors and total FIM at 1 month improved in patients with Pusher syndrome and unilateral spatial neglect in the deviation of variability errors group. Previous studies have reported delayed recovery of function and ability in patients with Pusher syndrome (Krewer et al., 2013; Pedersen et al., 1996) and decreased ADLs by severity (Babyar et al., 2017; Danells et al., 2004). For this reason, patients in the deviation of variability errors group had SPV directional errors within the normal range, decreased SPV variability errors, and improved total FIM at 1 month. These changes occurred because of the influence of the starting position measurement on the judgment of body verticality among patients. However, when the deviation of SPV directional errors was small, learning self‐body posture control for correcting SPV variability errors might be possible. Furthermore, Danells et al. (2004) reported that pushing behavior, measured by SCP, decreased significantly from 1 week to 3 months. In this study, the recovery of pushing behavior lasted approximately 2 months from the onset of the disease, suggesting that SPV variability errors were corrected, and total FIM was recovered (Danells et al., 2004).

In addition, patients with Pusher syndrome with USN have increased SPV‐EO variability errors due to directional attention disorder (Sawa et al., 2022; Corbetta & Shulman, 2011; Karnath, 2007). In this study, the SPV directional errors were within the normal range, which may have facilitated the improvement in the total FIM.

No correlation was found between the initial SPV‐EO variability errors and total FIM after 1 month in patients with Pusher syndrome and USN in the deviation of variability errors group; however, a correlation was observed between the initial SPV‐EO variability errors and total FIM after 1 month in patients with Pusher syndrome and USN in the deviation of both directional and variability errors group. The correlation was moderately negative (*r *= −0.57, *p < *.05) (Cohen, 1988), and positively associated with pre‐FIM (*r *= 0.87, *p < *.01). This result is similar to the outcomes reported in another study by Bonan et al. (2006), who found a negative association between SVV and ADLs, suggesting that SPV‐EO may also affect the total FIM. Therefore, a decrease in SPV‐EO variability errors may have contributed to an increase in the total FIM and an improvement in ADLs after 1 month.

The prognostic factors in postfunctional independence measures of PB and USN in all groups were initial FIM and initial SPV‐EO variability errors. The predicted equation was as follows: post‐FIM = 0.79 initial FIM + (−2.05) initial SPV‐EO variability errors + 42.79 (*R =* 0.755, *R* ^2^ *=* 0.570, *F* value* =* 4.861, *p = *.036). SPV‐EO variability errors and initial FIM total score influenced FIM after 1 month. This finding may suggest that the integration of two factors, somatosensory and visual, influences ADLs independence after 1 month during the recovery of patients with stroke with Pusher syndrome and USN. An assessment of modalities involved in vertical cognition and balance, which affect fall risk, may help stroke patients to maintain and improve their ADLs.

This study had some limitations. First, this study did not evaluate long‐term outcomes in patients with stroke and Pusher syndrome. Second, mortality rates were no evaluated. Third, this was a single‐center study, which may be affected by selection bias, despite including consecutive cases. Fourth, we included cases with different trajectories, thus the number of days since disease onset varied. Further studies are required to validate the present findings and to evaluate other populations.

## 5 CONCLUSIONS

No correlation was found between the initial SPV with eyes opened variability errors and total FIM after 1 month in patients with Pusher syndrome and USN in the normative range of SPV directional errors and without variability errors. However, a correlation was observed between the initial SPV with eyes opened variability errors and total FIM after 1 month in patients with Pusher syndrome and USN without normative SPV directional errors and variability errors in the deviation of both directional and variability errors group.

SPV with eyes opened variability errors and initial FIM score may influence the independence of ADLs after 1 month during the recovery of patients with stroke with Pusher and USN without normative SPV directional and variability errors in the deviation of both directional and variability errors group.

## FUNDING

The study did not have any role in the design and collection, analysis, and interpretation of data and in writing the manuscript. No funding was received for this work.

## CONFLICT OF INTEREST STATEMENT

The authors report no competing interests.

### ETHICS STATEMENT

This study was performed following the Declaration of Helsinki and approved by the Sonodakai Ethics Committee (No. 141). Furthermore, all participants provided written informed consent.

### PEER REVIEW

The peer review history for this article is available at https://publons.com/publon/10.1002/brb3.3001.

## Data Availability

University Hospital Medical Information Network (UMIN) Center: 000049806.
